# Bloomingdale Asylum

**Published:** 1848-05

**Authors:** 


### BLOOMINGDALE ASYLUM.

We have received the twenty-seventh *Annual Report of the Bloomingdale Asylum for the Insane*, by Pliny Earl, M. D., the physician of the asylum, which exhibits a favorable condition of things in the instiution. The whole number of cases in the asylum in the course of the year was 274, of which number 58 were discharged cured, 17 much im proved, 23 improved, and 18 unimproved—in all 116. Four of those who were much improved, and two who were improved, progressed rapidly to entire recovery after their return home—making the total number cured 64. A meteorological register for the year 1847 is appended to the report.

